# Spectral entropy of early-life distress calls as an iceberg indicator of chicken welfare

**DOI:** 10.1098/rsif.2020.0086

**Published:** 2020-06-10

**Authors:** Katherine A. Herborn, Alan G. McElligott, Malcolm A. Mitchell, Victoria Sandilands, Brett Bradshaw, Lucy Asher

**Affiliations:** 1 School of Biological and Marine Sciences, University of Plymouth, Plymouth, UK; 2 Centre for Research in Ecology, Evolution and Behaviour, Department of Life Sciences, University of Roehampton, London, UK; 3 Department of Animal and Veterinary Sciences, SRUC, Easter Bush, Midlothian, UK; 4 Department of Agriculture, Horticulture and Engineering Sciences, SRUC, Easter Bush, Midlothian, UK; 5 School of Natural & Environmental Sciences, Newcastle University, Newcastle upon Tyne, UK

**Keywords:** animal welfare, bioacoustics, iceberg indicator, precision livestock farming, *Gallus gallus domesticus*

## Abstract

Chicks (*Gallus gallus domesticus*) make a repetitive, high energy ‘distress’ call when stressed. Distress calls are a catch-all response to a range of environmental stressors, and elicit food calling and brooding from hens. Pharmacological and behavioural laboratory studies link expression of this call with negative affective state. As such, there is an *a priori* expectation that distress calls on farms indicate not only physical, but emotional welfare. Using whole-house recordings on 12 commercial broiler flocks (*n* = 25 090–26 510/flock), we show that early life (day 1–4 of placement) distress call rate can be simply and linearly estimated using a single acoustic parameter: spectral entropy. After filtering to remove low-frequency machinery noise, spectral entropy per minute of recording had a correlation of −0.88 with a manual distress call count. In videos collected on days 1–3, age-specific behavioural correlates of distress calling were identified: calling was prevalent (spectral entropy low) when foraging/drinking were high on day 1, but when chicks exhibited thermoregulatory behaviours or were behaviourally asynchronous thereafter. Crucially, spectral entropy was predictive of important commercial and welfare-relevant measures: low median daily spectral entropy predicted low weight gain and high mortality, not only into the next day, but towards the end of production. Further research is required to identify what triggers, and thus could alleviate, distress calling in broiler chicks. However, within the field of precision livestock farming, this work shows the potential for simple descriptors of the overall acoustic environment to be a novel, tractable and real-time ‘iceberg indicator’ of current and future welfare.

## 1. Introduction

An ‘iceberg’ welfare indicator is a single marker that covaries with a range of physical, behavioural and emotional welfare concerns [1]. The ‘distress call’ is a repetitive, high energy vocalization made by young chickens (*Gallus gallus domesticus*) when stressed [2], which could be a candidate iceberg indicator. Its association with negative emotional (affective) states has been pharmacologically validated in laboratory studies with anxiolytics and antidepressants [3]. Contexts eliciting distress calling are found also to elevate physiological stress markers including corticosterone and interleukin-6 [3,4], and negatively impact on cognitive indicators of mood [5,6]. Distress calling is so reliably triggered by social isolation that it has been proposed as a screening assay for drug development [7]. In early life, chicks are dependent on the hen thermally and for foraging, and the function of the call is to attract attention and elicit ‘food calls’ from the hen [8]. As such, this communication with the absent hen may be a sensitive indicator of emerging environmental concerns affecting commercially reared chicks. Distress calling appears not to be triggered directly by acute, startling stimuli (loud noise or electric shock [9]; air puff [10]), but rather discomfort or risky contexts: heat stress [11,12], cold stress [13], maternal signalling of threat [14], high density or food/water restriction (manipulated simultaneously [15]) or social isolation [2,9]. More generally, as young chicks are moved from the hatchery to the unfamiliar rearing environment, latency to settle and find resources and comfort is of critical welfare consideration [16], and distress calling is triggered by environmental unfamiliarity [9,13]. There is good *a priori* evidence from laboratory studies therefore, that distress call monitoring may be a real-time and animal-centred marker of both emotional state and environmental stressors on poultry farms.

In chickens, early-life welfare constraints often predict late-life welfare concerns [17]. One mechanism linking life stages is that stress is energetically costly: altered scope to invest in concurrent growth and immune function at critical points in development has consequences for downstream phenotype and mortality risks [18]. In broilers for example, elevating corticosterone to mimic stress exposure suppresses weight gain and heart development, increases oxidative damage and shifts investment from musculoskeletal growth and into fat deposition and antioxidant production [19,20]. Stress-induced activation of the developing neuroendocrine system in early life can also up- or downregulate later life stress-responsiveness [21]. In some contexts, though, this 'phenotypic programming' may enable animals to cope better when the same stressors are re-encountered (e.g. cold stress [22]; social and resource deprivation [23]). To validate distress calling as a welfare indicator therefore, it is important to explore links to welfare in both current and future life stages.

Precision livestock farming is the application of the principles of process engineering to the management of livestock [24]. Several recent studies propose automated acoustic approaches for monitoring poultry welfare, health and productivity in real time, in order to promote earlier husbandry interventions [25]. For example, acoustic tools have been proposed to monitor growth [26], feed intake [27], infectious bronchitis [28,29], necrotic enteritis [30], thermal comfort [31,32] and disturbance [32]. Most use machine learning approaches for classification, with algorithms trained on group-level recordings of flocks differing in health or stress exposure. Typically, classification requires several combined markers, but when a single acoustic feature shows a pronounced, directional change with a welfare or productivity concern, then statistical approaches are possible. For example, the lowering in fundamental frequency that occurs with increasing vocal tract length may be used to monitor growth in broilers [26]. However, while these top-down approaches have proven utility, regarding welfare there is often an *a priori* reason to start with a specific type of vocalization. Chickens have over 20 context-specific calls [33], thus ‘eavesdropping’ on the right sound could reveal functional (e.g. vigilance [34]; feeding [35]) or emotional states (frustration [36]; anticipation [37]). Moreover, specific sounds are linked to thermal discomfort [31] and pain [38], and certain reflexive sounds to respiratory diseases [29].

Automated detection of specific sounds is computationally challenging in a commercial environment with often thousands of overlapping calls and background machinery noise. For rare and acoustically distinctive sounds, such as coughing in calves, a bottom-up approach is possible, with classification algorithms trained on the sound specifically rather than the group disease status [39]. Distress calling is not rare: thousands of chicks may call simultaneously. However, it is distinctive: young (up to 6-day-old) chicks have a relatively simple vocal repertoire with greater than 90% of calls one of 4 relatively quiet ‘contact calls’ and just 1 common, loud (up to 92 dB) and repetitive distress call [2]. We anticipated that simultaneous calling would significantly alter the overall acoustic environment. As a catch-all response to a range of environmental stressors, we hypothesized that distress calling may be an iceberg welfare indicator. As such, our objective was to test whether a simple, statistical approach could be used to monitor this specific call type in a commercial broiler farm.

Using recordings of the first 4 days of placement in 12 flocks, we explored (1) whether house-level acoustic parameters changed in proportion to the intensity of flock-level distress calling, as determined from a manual distress call count. As a behavioural response to stress, we expected (2) that distress calling should be sensitive to changes in flock behaviour linked to welfare. As distress calls elicit food calling and brooding from the hen, we expected chicks to distress call more when using resources and when exhibiting tight clustering as a behavioural sign of cold stress [13]. We also expected distress calling to decline with age as chicks become both thermally independent and more settled within their environment [9,13]. Finally, lower than expected daily weight gain and high intra-flock mortality can indicate suboptimal environmental conditions or health in broilers [40]. To be an iceberg indicator, we, therefore, hypothesized that (3) this behavioural indicator of early-life stressor exposure would also predict low weight gain and high mortality. These parameters were measured at flock level on the day following acoustic recording and again toward slaughter age to test the short- and long-term predictive power of early-life distress call monitoring.

## 2. Methods

See electronic supplementary material where indicated for additional detail on datasets and methodological decisions.

### 2.1. Field data collection

Data were collected from 12 commercial Ross 308 mixed-sex flocks (25 090–26 510 chicks placed per flock). These constituted three consecutive placements into four houses (1314–1322 m^2^) on one farm, between 3 November 2017 and 15 March 2018.

**Figure 1.**
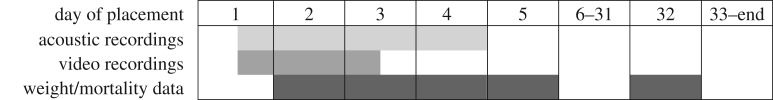
Schematic of acoustic, video and weight/mortality data collection days per flock.

See figure 1 for the schedule of data collection. To capture early-life distress calling, acoustic recorders ran for 4 days following placement. ‘Day 1’ commenced with arrival from the hatchery (10.00–16.00, mean 14.15, variation due to commercial constraints on delivery time) and ended at midnight, and days 2–4 were 24 h cycles thereafter. For each flock, a 9 mm diaphragm condenser microphone was positioned centrally in the front right quadrant of the house at 70 cm above ground height (beyond reach of chicks), 40 cm from the end of a perch and 1 m equidistant to a feeder line and drinker line. Recordings sampled at 44.1 kHz were gathered at a 1 min/10 min interval throughout the day (i.e. 144 recordings per flock per day) using an Arbimon Acoustic™ recorder (Sieve Analytics, San Juan, Puerto Rico). To explore within-house consistency in acoustic parameters, a second recorder was installed above the same arrangement of features centrally in the rear left quadrant of two houses (*n* = 6 flocks).

To analyse correlations between behaviour and distress calling, chicks were videoed from day 1 to 3. For each flock, a GoPro 5™ camera with Blink™ time-lapse controller (Camdo Solutions, California, USA) was positioned at 240 cm above the ground over the microphone (parallel with lighting rigs). Videos were collected at a 1 min h^−1^ interval. Two video feeds were lost at 13 h, with the remainder running 30–49 h (mean 40.6 h), generating 493 videos in total. All equipment was installed prior to placement and retrieved at house clearance.

### 2.2. Automated acoustic data extraction

One-minute recordings were characterized using R v. 3.5.1 [41] using the packages TuneR [42] (function: readWave) and Seewave [43] (functions: ffilter, meanspec and specprop). The mean frequency spectrum was obtained using short-time Fourier transform with a 512 sample, non-overlapping Hanning window. Three datasets were produced with different band-pass filters applied to the same files: (1) ‘unfiltered’; (2) ‘high-pass’ filtered above 2750 Hz to remove low frequency fan and heater noise without encroaching on frequency ranges where most energy in distress vocalizations is distributed for 1- to 5-day-old chicks (above 2756 Hz [44]); (3) additionally low-pass filtered above 5000 Hz to encompass the frequency range where most energy in distress calls is distributed (2756–4307 Hz [44]; ‘call region’). For each recording in each dataset, 12 parameters were extracted. Nine describe average frequency: mean, median, standard deviation and standard error of the mean, dominant frequency (frequency of maximum amplitude), 25th and 75th quartiles (below/above which 25% of energy in the spectrum is found) and the interquartile range (75th–25th quartile). Four describe the shape of the power spectrum (where *x* = frequencies, *y* = relative amplitude of the *i* frequency, *N* = number of frequencies): centroid (sum(*x* × *y*)), skewness (sum((*x*-mean(*x*))^3^)/(*N* − 1)/sd^3^), kurtosis (sum((*x*-mean(*x*))^4^)/(*N* − 1)/sd^4^) and spectral entropy (−sum(*y*log*y*)/log(*N*)) [43].

### 2.3. Manual acoustic data extraction

To validate the automated acoustic parameters as measures of distress calling, distress calls were manually counted in 283 1-min acoustic recordings using Praat [45] (see electronic supplementary material methods). A distinctive shape (brief ascending then prolonged descending frequency modulation, 100–250 ms [2]) allowed distress calls to be identified in spectrograms. To capture any acoustic differences in distress calls that may occur with welfare status or age in the validation set, 23–26 files were selected per flock from day 1 to 4, at 2 h intervals on day 1 and 4 h intervals thereafter.

### 2.4. Video data extraction

Video data were used to analyse correlations between distress calling and flock-level behaviour. To explore the effects of behaviour at different distances to the microphone on acoustic recordings, three 2 m^2^ square areas were identified per video: a ‘Microphone’ square with the microphone in the centre, and two adjacent ‘Surrounding’ squares. Each encompassed 2 (1 full and 2 half) feeder pans and 10 nipple drinkers, where the feeder and drinker line demarcated the opposing sides. We could not reliably follow individuals, so to avoid psuedoreplication within videos, a count of chicks (‘Total chicks’, see electronic supplementary material methods) and spatial distribution were recorded once per video, at time 0, and activity, foraging and drinking behaviour were recorded in the first 10 s. ‘Distribution’ was categorized: 1 (spaced apart, chicks had 0 or 1 chick within 1 body-length), 2 (2+ chicks within 1 body-length, forming small clusters), 3 (large clusters in physical contact and the central chick greater than 2 body-lengths from the cluster edge). Category 3 is analogous to distributions used by stockpersons to identify cold stress [16]. ‘Activity’ was categorized from 0–3 as follows: 0 (0–5% of chicks moving), 1 (less than 50% of chicks moving), 2 (greater than 50% of chicks moving). The occurrence of ‘Large-scale movements' (greater than 50% of chicks moving between rather than within squares) was a binary variable scored for the whole minute (yes/no). ‘Drinking’ was a count of chicks observed using nipple drinkers. As feed was scattered on the litter as well as available in hoppers, ‘foraging’ was a count of chicks observed either with heads down/scratching or directly pecking at feeder pans (see electronic supplementary material methods). Numeric variables: foraging (Pearson's *r* = 0.32, *t* = 7.42, *p* < 0.0001), drinking (*r* = 0.43, *t* = 10.64, *p* < 0.0001) and total chicks (*r* = 0.27, *t* = 6.10, *p* < 0.0001), were significantly correlated across and averaged for the two Surrounding squares. Distribution and Activity were classified across the two Surrounding squares combined: classifications which correlated also with behaviour in the broader field of view (see electronic supplementary material methods). Due to the overhead view, individuals distress calling could not be identified in videos to explore their behaviour specifically.

### 2.5. Welfare and productivity data

To test whether early-life distress calling predicted immediate and future weight or mortality, farm productivity data were collated for the days following acoustic measurements (days 2–5 of placement) and day 32. Mortality (birds found dead, excluding culls) was collated from stockperson records. Average bird weight was provided by the producer, from a commercial algorithm that used data collected continuously from two platform balances (Fancom Automatic Poultry Weighing System; Leuven, Belgium) in each house. Because data were collected on a commercial farm, ages for house thinning (25–30% flock slaughtered, 33–34 days) and clearance (36–39 days) varied as required to optimize productivity. For consistency, endpoint data were therefore gathered at 32 days.

### 2.6. Statistical methods

The best automated proxy of manual distress call counts was assessed (1) by correlation coefficient. Spearman's correlations were used due to skew towards low manual call counts. And (2), using a random forest approach for parameter selection (R random Forest [46]). The model compared 2000 trees, each composed of subsets of 12 variables to reduce impacts of collinearity in parameter selection. Parameter ‘importance’ in this approach is the difference in mean squared error with random permutation of each variable, normalized by the standard deviation in those differences.

For the parameters with the strongest correlation, (3) a linear mixed model (LME) was fitted for each with manual call count as the dependent, flock as a random effect to account for repeated measurements, and the interaction of (day of placement (factor, 1–4)× the acoustic predictor) as independent variables. A likelihood ratio test (LRT) between models with and without the interaction was used to assess whether the slope of the relationship between the acoustic predictor and manual call count was age dependent. From these three approaches, the strongest single parameter (termed the ‘acoustic predictor’ for the remainder of the methods) was identified.

Distribution was markedly more variable and distress calling more prevalent on day 1 (see electronic supplementary material methods), so separate models investigated correlations between the acoustic predictor and day 1 (115 time-matched videos, 12 flocks) versus day 2–3 behaviour (378 videos, 10 flocks). In both analyses, an LME fitted with maximum-likelihood controlled for repeated measurements per flock as a random effect. Total chicks, distribution, foraging, drinking and activity for microphone and surrounding squares and the parameter large-scale movements were independent variables. In the day 1 model, hours from placement was also included as a covariate. On day 1, activity in surrounding squares was predominantly category 2 (106 of 115 records), thus captured by the large-scale movement parameter (effectively activity category 2 versus large-scale movements), so was omitted from the model. The day 2–3 model included day of placement as a factor (2 or 3, categorical). Microphone and surrounding square total chick counts were retained in models to control for numbers near the microphone, but models were otherwise simplified by backwards stepwise regression, using LRT to compare consecutive models (threshold *p* < 0.05) until only significant variables and chick counts remained.

Finally, we tested whether the acoustic predictor, expressed as an average of the recordings collected per flock per day, was predictive of future mortality and weight gain. On days 2–4, this was an average of 144 recordings over 24 h, and on day 1, due to variation in placement time, of 42 recordings collected over h 1–8 of placement (see electronic supplementary material methods). In the first pair of models, the response variables were average bird weight and proportion flock mortality in the next day. There were four datapoints per flock (day 2, 3, 4 and 5 mortality or weight), thus LMEs were used to control for repeated measurements. The main effects and interaction of (acoustic parameter × age) were independent variables. Mortality was logged to improve model fit. In the second pair of models, linear models were constructed with the response variable of either average bird weight or % flock mortality of the starting flock by 32 days. In these models, there were 12 data points (one end-point measure per flock), and four independent variables: the average of the acoustic predictor for day 1, 2, 3 and 4. Models were simplified by stepwise backward regression until only significant variables remained.

Sound was off during video data extraction, and acoustic, video and welfare data were compiled separately so that the researchers would remain blind to outcomes during manual data extraction.

## 3. Results

### 3.1. Validation of house-level acoustic parameters

**Figure 2.**
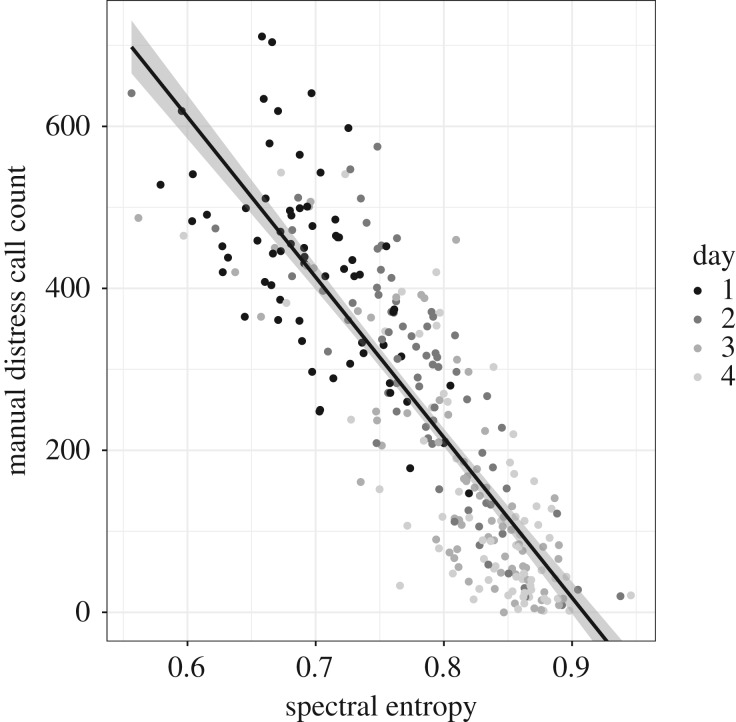
Relationship between spectral entropy extracted from high-pass filtered recordings and manual count of distress calls per minute; point colour indicates day of placement, shaded area indicates confidence interval.

Across both correlation (*ρ*: −0.88) and random forest approaches, spectral entropy proved the best acoustic predictor of manual distress call count, where negative values indicate high distress call counts (table 1 and figure 2). Paired T-tests of Spearman's *ρ* values (made positive) between unfiltered and filtered data indicate that strength of correlation was significantly improved by high-pass filtering (*t* = 2.29, *p* = 0.043), but only marginally by additional low-pass filtering (unfiltered compared to call region data: *t* = 1.80, *p* = 0.06). While high-pass filtered spectral entropy (henceforth spectral entropy) ranked highest, four other parameters shared an equivalent Spearman's *ρ* and were selected next in random forest models: interquartile range, 75% quartile, the centroid and the mean of the frequency distribution.

**Table 1.** Correlations between manual call count and acoustic parameters ranked by Spearman's correlation and importance in random forest model, where importance of a variable is expressed as the % increase in mean squared error if a variable is randomly permuted.

parameter	filter	Spearman's correlation		random forest	
		*ρ*	*rank*	*importance*	*rank*
spectral entropy	high pass	−0.88	1	28.73	1
interquartile range	high pass	−0.88	4	28.60	2
75th quartile	high pass	−0.88	3	28.56	3
mean	high pass	−0.88	=2	26.20	4
centroid	high pass	−0.88	=2	25.87	5
standard error of the mean	high pass	−0.71	6	20.48	6
25th quartile	call region	0.44	18	18.49	7
standard deviation	call region	−0.62	=8	18.05	8
centroid	unfiltered	0.15	=26	17.50	9
median	high pass	−0.71	=5	17.26	10
standard deviation	high pass	−0.71	=5	16.99	11
standard deviation	unfiltered	−0.64	=7	15.72	12
median	call region	−0.62	=8	15.70	13
median	unfiltered	−0.64	=7	15.05	14
25th quartile	unfiltered	0.57	9	15.00	15
mean	unfiltered	0.15	=26	14.85	16
25th quartile	high pass	−0.29	24	14.10	17
dominant	call region	0.57	=10	13.84	18
dominant	high pass	0.57	=10	13.76	19
kurtosis	call region	0.35	23	13.56	20
kurtosis	high pass	0.38	21	13.03	21
skewness	high pass	0.41	20	12.91	22
kurtosis	unfiltered	−0.56	11	12.81	23
standard error of the mean	unfiltered	0.52	13	12.66	24
interquartile range	unfiltered	−0.48	16	12.62	25
standard error of the mean	call region	0.10	28	12.23	26
dominant	unfiltered	0.54	12	12.07	27
spectral entropy	call region	−0.45	17	11.88	28
skewness	call region	0.36	22	11.83	29
centroid	call region	−0.01	30	10.85	30
spectral entropy	unfiltered	−0.42	19	10.78	31
interquartile range	call region	−0.51	14	9.86	32
mean	call region	−0.01	29	9.80	33
skewness	unfiltered	−0.49	15	9.56	34
75th quartile	unfiltered	0.11	27	9.39	35
75th quartile	call region	−0.19	25	8.81	36

Spectral entropy extracted from time-matched recordings was slightly higher at the front than rear of houses (paired T-test: *t* = 2.29, d.f. = 2584, *p* = 0.023). The difference was small (mean difference 0.002 ± C.I. 0.0002–0.0003) and time-matched data were strongly correlated (Pearson's correlation: *t* = 82.1, d.f. = 2583, *p* < 0.0001, *R* = 0.85 ± C.I. 0.84–0.86; electronic supplementary material, figure S1).

While as expected, distress call rate declined significantly with age, the slope of spectral entropy with manual distress call count was age-independent (table 2*a*). By contrast, the slopes of the next highest ranked parameters, high-pass filtered interquartile range and 75th quartile, and manual distress call count were age dependent (electronic supplementary material, table S1 and figure S2).

**Table 2.** Linear mixed models of the relationship between spectral entropy and (*a*) manual distress call count (*N* = 283/12 flocks), and (*b*) day 1 (*n* = 115/12 flocks) and (*c*) days 2–3 (*N* = 378/10 flocks) behaviour and distribution in the 2 m^2^ around the microphone versus the surrounding area. In model (*a*), a likelihood ratio test indicates no significant difference in slope with day, thus an estimate of −0.0003 ± 0.00001 change in spectral entropy per additional distress call can be applied across the age range sampled to describe the changes in distress calling with 1 unit change in significant parameters of models (*b*) and (*c*). Flock was a random effect in all models.

	value	s.e.	d.f.	*t*-value	*p*-value	LRT *χ*^2^
(*a*) *model: age effects*						
intercept	1769.01	65.15	267	27.15	<0.0001	
spectral entropy	−1927.34	91.43	267	−21.08	<0.0001	
day – 2	26.74	15.80	267	1.69	0.092	
day – 3	−36.07	17.17	267	−2.10	0.037	
day – 4	−34.23	18.40	267	−1.86	0.064	
spectral entropy × day					0.23	4.36
(*b*) *model: day 1 behaviour*						distress calls
intercept	0.776	0.014	97	53.64	<0.0001	
surrounding – drinking	−0.003	0.001	97	−4.66	<0.0001	+10 (±3)
surrounding – foraging	−0.001	0.000	97	−4.00	0.0001	+3.3 (±0.3)
microphone activity – 1	−0.027	0.012	97	−2.15	0.034	+90 (±40)
microphone activity – 2	−0.050	0.012	97	−4.12	0.0001	+167 (±40)
surrounding total chicks	0.000	0.000	97	1.28	0.020	−0.46 (±0.3)
microphone total chicks	0.000	0.000	97	0.58	0.57	
(*c*) *model: days 2–3 behaviour*						
intercept	0.814	0.012	358	68.20	<0.0001	
surrounding distribution – 2	−0.006	0.009	358	−0.68	0.50	
surrounding distribution – 3	−0.025	0.010	358	−2.50	0.013	+116 (±33)
microphone distribution – 2	0.016	0.008	358	2.03	0.044	−53 (±27)
microphone distribution – 3	0.002	0.011	358	0.17	0.87	
surrounding activity – 1	−0.025	0.008	358	−3.05	0.0025	+83 (±27)
surrounding activity – 2	−0.019	0.014	358	−1.44	0.15	
microphone activity – 1	−0.003	0.008	358	−0.36	0.72	
microphone activity – 2	−0.027	0.013	358	−2.04	0.042	+90 (±43)
surrounding – total chicks	0.000	0.000	358	−0.10	0.92	
microphone – total chicks	0.000	0.000	358	1.44	0.15	

### 3.2. Behaviour

On day 1, low spectral entropy (high distress calling) was correlated with parameters describing resource access and the number or activity of chicks (table 2*b*). Low spectral entropy occurred when levels of foraging and drinking in Surrounding squares were high. Spectral entropy was lower when birds were more active in the Microphone square, which may simply reflect that birds were awake thus able to call. It was also low when total chicks in Surrounding squares was low, though unrelated to total chicks in the Microphone square. Other variables were non-significant and removed from the day 1 model.

On days 2–3, low spectral entropy (high distress calling) instead correlated with parameters describing chick spatial distribution (table 2*c*), and behaviour in Microphone versus Surrounding squares had contrasting affects. Spectral entropy was lower when distribution in Surrounding squares occurred in large (category 3) clusters rather than spread out (category 1). However, spectral entropy was higher when the chicks in the Microphone square were distributed in intermediate (category 2) clusters. Like the day 1 model, spectral entropy was relatively low when chicks in the Microphone square were active. However, spectral entropy was also lower when some but not all chicks (5–50%) were active in Surrounding squares. Neither Microphone nor Surrounding total chick counts predicted spectral entropy on days 2–3, which may reflect a more even distribution in the house than on day 1.

### 3.3. Predictive models of weight and mortality

Spectral entropy was right-skewed, thus a median value per flock per day was calculated to test correlations with weight and mortality. The relationship of spectral entropy and average bird weight in the next day was age-dependent: positive on day 1 (high distress calling: low weight), flat on day 2, then increasingly positive through days 3 and 4 (table 3). The relationship with log(% flock mortality) in the next day was independent of age: low spectral entropy (high distress calling) predicted high mortality (table 3).

**Table 3.** Minimum adequate linear mixed models explaining variation in (*a*) proportion of flock mortality and (*b*) average bird weight on the day following acoustic recording. Flock ID (*n* = 12) was included as a random effect.

parameter	value	s.e.	d.f.	*t*-value	*p*-value
(*a*) *model: log(proportion flock mortality) in the next day*					
intercept	1.240	0.817	35	1.52	0.14
median spectral entropy	−3.183	1.030	35	−3.09	0.0039
(*b*) *model: average bird weight (g) in the next day*					
intercept	0.013	0.019	29	0.66	0.52
median spectral entropy	0.072	0.028	29	2.58	0.015
day – 2	0.061	0.019	29	3.24	0.003
day – 3	0.069	0.023	29	3.01	0.0054
day – 4	−0.032	0.027	29	−1.18	0.25
median spectral entropy × day – 2	−0.064	0.026	29	−2.43	0.022
median spectral entropy × day – 3	−0.055	0.030	29	−1.80	0.082
median spectral entropy × day – 4	0.092	0.034	29	2.68	0.012

**Figure 3.**
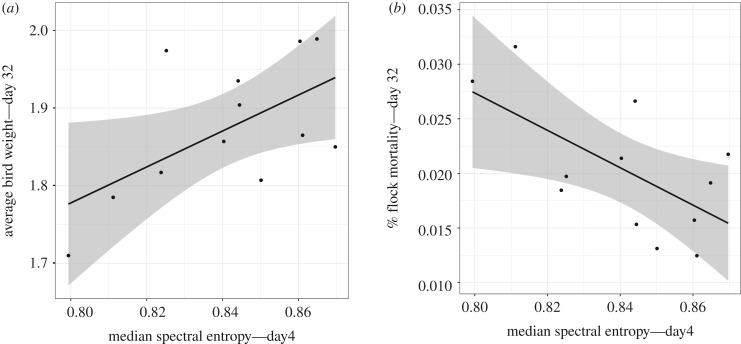
Relationship between median spectral entropy extracted from high-pass filtered recordings on day 4 of placement and (*a*) average bird weight (kilogram) and (*b*) % flock mortality by day 32 of placement. Shaded area indicates confidence interval.

Both a low average bird weight (slope 2.32 ± 1.00, *t* = 2.31, *p* = 0.043) and high % flock mortality (slope −0.171 ± 0.067, *t* = −2.57, *p* = 0.028) on day 32 were predicted by low median spectral entropy (high distress calling) on day 4 (figure 3).

## 4. Discussion

Automated monitoring of livestock has great potential to provide real-time warnings of emerging welfare concerns [47]. Here, spectral entropy, high-pass filtered to remove low frequency machinery noise, proved a simple, linear correlate of manual distress call count in the first 4 days of placement (Spearman's *ρ*: −0.88). Consistent with expectation, distress calling decreased with age, and this was captured by increases in spectral entropy. Ecologically, distress calling is a catch-all response to a range of acute stressors and interestingly, it was linked to different behaviours with age: feed/water use on day 1 versus distribution and activity patterns on days 2–3. Most importantly, it predicted future weight and mortality, not only into the next day but toward the end of production (day 32). Together, this is strong evidence that it is both possible and relevant to monitor chick distress calling using a whole-house measurement of this single acoustic parameter.

Spectral entropy describes the complexity of a system; in acoustic data, low values reflect tonal sounds while high values approach white noise. Compared to other call types, distress calls are loud (up to 98 dB) and expressed in repetitive series [2], thus it is unsurprising that shifts in overall acoustic environment reflect changes predominantly in this call. On day 1, the low spectral entropy reflects hundreds or thousands of similarly sized and hence pitched chicks calling in unison. However, on day 2–3, changes in spectral entropy that occurred with changes in flock-level behaviour were consistent with relatively few additional birds calling. That the relationship with manual distress call count was linear across ages and counts suggests that this approach is scalable to the decline in call rates with age. Moreover, sensitivity to low numbers may allow ‘first responders’ to welfare concerns to be detected, where individual chickens are expected to differ in both environmental sensitivity and physiological stress responsiveness [48].

Several other parameters were almost equally well correlated to manual distress call count: frequency centroid, mean frequency and the upper and interquartile ranges of frequency all had a Spearman's *ρ* of 0.88. Spectral entropy proved more tractable than parameters ranked next by the random forest approach, as the slope of the correlation with manual call count was independent of age. This is likely because low spectral entropy captures the presence rather than specific frequency of a peak in the power distribution, where call frequency is expected to shift as chicks grow [26]. Monitoring a specific call type in adult chickens, which have a complex vocal repertoire (greater than 20 context-specific calls [33]), requires a combination of acoustic parameters for accurate classification. This capacity to use single parameters to capture distress calling in chicks, and redundancy between them, offers scope to select parameters to either generalize across or specialize within ages. Or indeed commercial contexts, where acoustics may vary among strains with different vocalization rate [49], flocks with different health status [50], or houses with different machinery noise [51].

For most of the acoustic parameters, high-pass filtering to remove low frequency machinery noise significantly improved strength of correlation with manual distress call count (e.g. for spectral entropy, high-pass *ρ* = −0.88, unfiltered *ρ* = −0.42). Conversely, while most power in distress calls occurs between 2756 and 4307 Hz [44], additionally low-pass filtering above 5000 Hz weakened correlations (e.g. for spectral entropy *ρ* = −0.45). We conclude that it is more important to retain relatively low power, higher frequency components of distress calls than to filter out high frequency background noise such as rustling or pecking that may be equivalent when chicks are and are not calling. As background noise may vary between farms, more sensitive filtering approaches that quantify and subtract it could be beneficial (e.g. [12,52]).

On day 1, chick distribution varied markedly. That spectral entropy was low (distress calling high) when there were low counts of chicks in the surrounding area suggests that the amplitude of distress calls may allow responses, potentially to social or thermal isolation, of even widely dispersed chicks to be captured. Consistency in behaviour within different areas in the videos and moreover between time-matched acoustic data collected in the front and rear of houses suggests behavioural synchrony within flocks. Therefore, a single Microphone appears sufficient to monitor house distress calls. However, there was a subtly higher rate of distress calling at the rear than front of the houses. Even within relatively homogeneous farm environments, individual chickens show distinct and consistent patterns of space use [53]. This raises the possibility that rear-of-house Microphones captured different ‘types’ of chick, either in microhabitats with different exposure to welfare concerns or subpopulations with different sensitivity to them. Future work is required to explore this spatial heterogeneity.

Early life adversity has profound consequences for late life phenotype and fitness [18]. Here, low distress calling on day 4 predicted high average bird weight and low cumulative mortality at 32 days (close to slaughter age). While in early life spectral entropy predicted next-day mortality independently of age, a strengthening in the predictive relationship with next-day weight occurred through days 1–4. Part of the call function is to elicit hen behaviour to help chicks locate resources [8]. The association between calling and feed and water use on day 1, therefore, may be explained by hunger or thirst, from deprivation during transport, unfamiliarity with resource distribution, and/or stress, which may increase these motivations [19,20,54]. However, distress calling should then reduce as chicks become familiar with and imprint on a context [9,13]. We suggest that continued distress calling on day 4 thus reflects either persistent or cumulative stress exposure or failure to settle. As such, day 4 may be a particularly sensitive period for predicting future welfare outcomes.

Different behaviours were associated with distress calling on days 2–3 than day 1. On days 2–3, spectral entropy was low (distress calling high) when chicks were spread out, where social and thermal isolation are expected to trigger distress calling [2,13]. However, it was also higher when chicks occurred in large, tightly packed clusters. This result mirrors an experimental study that found a correlation between ‘swarming’ behaviour that is associated with cold stress [16] and overall vocalization amplitude [11]. Thus, cold stress may be one trigger of calling. Interestingly, distress calling on days 2–3 was also high when only some (5–50%) chicks were active, compared to none or all. It was also low when chicks occurred in small groups near the microphone, rather than singly or in dense clusters. In nature, behavioural synchrony allows siblings to optimize brooding and foraging opportunities, thus an asynchronously active chick that either disturbs resting individuals or is seeking others may be another source of distress calls. By contrast, large-scale movements that may indicate stockperson or other disturbance in the barn were not associated with distress calling. These events may be more analogous to predation events, where calling poses a detection risk, and previous studies report no link between distress call rate and acute, startling events [9,10]. Importantly, that chicks may not distress call during stockperson checks indicates a need for automated, background monitoring.

As part of a precision livestock farming system, automated detection of welfare concerns should trigger interventions [25]. The short lifespan and high density of most commercial broiler flocks mean that early life warnings of any health or welfare compromise would be particularly advantageous. To date, much automated monitoring has focused on the use of video data. In chickens, movement patterns are intuitively sensitive to lameness and foot health (e.g. [55]), but also subtle, flock-level behavioural patterns that indicate either infection with or susceptibility to bacterial diseases [56]. While image analysis is undoubtedly valuable, where acoustic correlates are identified, audio files are smaller and thus easier, cheaper and quicker to transfer, process and store. Moreover, they may be preferable for farmers due to security and anonymity concerns, but particularly if parameter extraction occurs in sensors to avoid storing raw recordings. To validate this potential iceberg indicator fully, future work is required to integrate spectral entropy into routine commercial data collection, to determine how well it generalizes across contexts and what sensitivity it offers for early warning within existing husbandry, productivity and welfare data collection. Here, 98 h of data were generated from 12 flocks on one farm. In more acoustically complex, natural environments, 120 h of continuous recording is recommended to capture a representative sample of sounds [57]. It will be important therefore to explore the generalizability of these findings across farms, and to establish baselines that incorporate age and time of day effects. Second, shifts in growth and mortality are an indicative but relatively crude measure of welfare that sum together many different concerns [40]. A wider array of welfare markers should be explored to understand the mechanisms linking distress calling to growth and mortality. Finally, triggers of distress calling must be identified. In one sense, the simplicity of the chick ‘vocabulary’ is a strength for automation: one call type acts as a catch-all for a range of social and environmental parameters [2]. However, this generality may mean that distress calling serves better as a real-time warning that ‘something’ is wrong rather than as a guide for directing specific interventions.

Distress calls are not unique to chickens. Lingle *et al*. [58] reviewed neonate calls triggered by isolation or capture in a diverse taxonomic array. They note the following recurrent features: a tonal sound with a chevron followed by a flat or (here) descending note, emitted in bouts, and with a higher fundamental frequency and amplitude than contact calls. This ubiquity may reflect conserved sensory processing pathways that underly parental separation among vertebrates [59]. Intriguingly, across species, exposure to distress calls can influence the emotional state of receivers [60], suggesting that distress calling may be not only a welfare indicator but a welfare concern. By developing and validating this simple approach for distress call monitoring, this study opens new avenues into welfare research within commercially relevant contexts.

## Supplementary Material

Supplementary Materials

## Supplementary Material

Dataset 1: Acoustic parameters

## Supplementary Material

Dataset 2: Behavioural data

## Supplementary Material

Dataset 3: Weight and mortality

## Data accessibility

Datasets for the analyses reported are provided as electronic supplementary material.
