# Supplementary material for: Spectral entropy of early-life distress calls as an iceberg indicator of chicken welfare
Source: J R Soc Interface. 2020 Jun 10;17(167):20200086. doi: 10.1098/rsif.2020.0086 (PMC7328393; doi:10.1098/rsif.2020.0086)
Supplement: Supplementary Materials [file rsif20200086supp1.docx]

**Spectral Entropy of early life distress calls as an iceberg indicator of chicken welfare: Supplementary materials**

Katherine A. Herborn_1*_, Alan G. McElligott_2_, Malcolm A. Mitchell_3_, Victoria Sandilands_4_, Brett Bradshaw_5_, Lucy Asher_5_

_1_ School of Biological & Marine Sciences, University of Plymouth, UK

_2_ Centre for Research in Ecology, Evolution and Behaviour, Department of Life Sciences, University of Roehampton, UK

_3_ Department of Animal & Veterinary Sciences, SRUC, UK

_4_ Department of Agriculture, Horticulture & Engineering Sciences, SRUC, UK

_5_ School of Natural & Environmental Sciences, Newcastle University, UK

*Corresponding author

SUPPLEMENTARY METHODS

The supplementary methods are predominantly as the main text but include additional detail on the datasets or data collection where required to explain methodological decisions.

**Field data collection**

Data were collected from 12 commercial Ross 308 mixed-sex flocks (25090-26510 chicks placed per flock). These constituted three consecutive placements into 4 houses (1314-1322m^2^) on one farm, between 03/11/2017 and 15/03/2018.

To capture early-life distress calling, acoustic recorders ran for 4 days following placement. ‘Day 1’ commenced with arrival from the hatchery (10:00-16:00, mean 14:15, variation due to commercial constraints on delivery time) until midnight, and days 2-4 were 24 h cycles thereafter. For each flock, a 9 mm diaphragm condenser microphone was positioned centrally in the front right quadrant of the house at 70 cm above ground height (beyond reach of chicks), 40 cm from the end of a perch and 1 m equidistant to a feeder line and drinker line. Recordings sampled at 44.1 kHz were gathered at a 1 min/10 min interval throughout the day (i.e. 144 recordings per flock per day) using an Arbimon Acoustic™ recorder (Sieve Analytics, San Juan, Puerto Rico; Aide et al., 2013). To explore within-house consistency in acoustic parameters, a second sound recorder was installed above the same arrangement of features centrally in the rear left quadrant of two houses (n = 6 flocks).

To analyse correlations between behaviour and distress calling, chicks were videoed from Day 1-3. For each flock, a GoPro 5™ camera with Blink™ time-lapse controller (Camdo Solutions, California, USA) was positioned at 240 cm above the ground over the microphone (parallel with lighting rigs). Videos were collected at a 1 min / h interval. Cameras ran for between 13-49 h before time-lapse failure (n = 1, 13 h), obstruction (n = 1, 13hr) or battery depletion (n = 10, range 30-49 h, mean 40.6 h), gathering in total 513 videos from days 1-3 of placement. Videos occurring during dark periods or during placement or stockperson walk-throughs were excluded, leaving 493 videos that could be time-matched to acoustic recordings to explore behavioural correlates of distress calling. All equipment was installed prior to placement and retrieved at house clearance.

**Automated acoustic data extraction**

One-minute recordings were characterised in R version 3.5.1 using the packages TuneR (Ligges, 2018, function: readWave) and Seewave (Sueur, 2018, functions: ffilter, meanspec and specprop). The mean frequency spectrum was obtained using short-time Fourier transform (STFT) with window size of 512 samples and non-overlapping Hanning window to reduce computational time for eventual automation. Three datasets were produced with different band-pass filters applied to the same files: 1)‘unfiltered’; 2) ‘high-pass’ filtered above 2750 Hz to remove low frequency fan and heater noise (‘high-pass’) without encroaching on frequency ranges where most energy in distress vocalizations is distributed for 1-5 day old chicks (> 2756 Hz; Fontana et al., 2016); 3) additionally low-pass filtered above 5000 Hz to encompass the frequency range where most energy in distress calls is distributed (2756-4307 Hz, Fontana et al., 2016; ‘call region’). For each recording in each dataset, 12 parameters were extracted. Nine describe the average frequency: mean, median, standard deviation and standard error of the mean, dominant frequency (frequency of maximum amplitude), 25^th^ and 75th quartiles (below/above which 25% of energy in the spectrum is found) and the interquartile range (75^th^ – 25^th^ quartile). Four describe the shape of the power spectrum (where x = frequencies, y = relative amplitude of the i frequency, N = number of frequencies): centroid (sum(x*y)), skewness (sum((x-mean(x))^3)/(N-1)/sd^3), kurtosis (sum((x-mean(x))^4)/(N-1)/sd^4) and spectral entropy (-sum(ylogy)/log(N); Sueur et al., 2008).

**Manual acoustic data extraction**

To validate the automated acoustic parameters as measures of distress calling, distress calls were manually counted in 283 1-min acoustic recordings. To capture acoustic differences in distress call rate that may occur with welfare status or age in the validation set, 23-26 files were selected per flock from day 1-4. To ensure high calling that occurred on day 1 was included, one recording after 1 h of placement (to ensure stockpersons were no longer in the barn) and then at 2 h intervals until midnight were selected, with an alternative sample at 23:00 where the 2 h interval would fall at 00:00. Recordings were selected at 00:00 and 4 h intervals per flock thereafter for days 2-4. All visible distress calls were counted in spectrograms using Praat (Boersma, 2001). A distinctive shape (brief ascending then prolonged descending frequency modulation over time, 100-250ms; Marx et al., 2001) allowed distress calls to be identified in spectrograms (Fig. S1). Calls were most visible when counted in 3.25-7.5 s bins with a 0.03 s window length and 70 dB dynamic range. Manual counting can be subjective regarding inclusion of faint, overlapping or partially obscured calls, and thus one observer (KH) counted all recordings, with a Pearson’s R of 0.98 in a subset of 106, 7.5 s recordings that were counted twice with the observer blind to first count. Moreover, the observer was blind to welfare, video and automated acoustic data during manual acoustic data extraction.

**Video data extraction**

Video data were used to analyse correlations between distress calling and flock-level behaviour. Individuals distress calling could not be identified in videos to explore their behaviour specifically, so flock-level behaviour was recorded. To explore the effects of behaviour at different distances to the microphone on acoustic recordings, three 2 m^2^ square areas were identified per video: a ‘microphone’ square with the microphone in the centre, and two adjacent ‘surrounding’ squares. Surrounding squares shared one edge with the microphone square and were either within the same or the next feeder-drinker line dependent on visibility through overhead obstructions (heaters, pipes etc). The squares encompassed 2 (1 full and 2 half) feeder pans and 10 nipple drinkers, where the feeder and drinker line demarcated the opposing sides of each square. Prior to placement, it is common practice to scatter food onto lengths of paper laid parallel to feeder/drinker lines through the barn. While the paper quickly becomes covered with litter and disintegrates, observation of continued foraging in these areas indicate that it takes > 3 days for either the scattered food to be fully depleted or for learned foraging on previously successful locations to cease. In the study farm, perches and sawdust bales were also provided for enrichment. Exact placement of perches, bales and paper strips varied slightly between squares. The % floor coverage of squares by bales and perches was between 0-5% each, with chicks not yet using and able to freely pass under the latter. The % floor coverage per square with paper was more variable, at 0-30%, but was comparable for local versus surrounding squares across flocks.

We could not reliably follow individuals, so to avoid pseudoreplication within videos, a count of chicks (‘Total chicks’) and spatial distribution were recorded once per video, at time 0, and activity, foraging and drinking behaviour were recorded in the first 10 s. ‘Distribution’ was categorized from 1-3 as follows: 1 (spaced apart, chicks had 0 or 1 chick within 1 body-length), 2 (2+ chicks within 1 body-length, forming small clusters), 3 (large clusters with chicks in physical contact and the central chick > 2 body-lengths from the cluster edge). Category 3 is analogous to distributions used by stockpersons to identify cold stress in the days following placement (Ross management handbook, 2018). ‘Total chicks’ could be inaccurate when chicks were tightly clustered or mobile, so was estimated to the nearest 10, with a Pearson’s correlation coefficient of 0.97 between this estimate and an accurate count in a subset of 100 videos. ‘Activity’ was categorized from 0-3 as follows: 0 (0-5% of chicks moving), 1 (< 50% of chicks moving), 2 (> 50 % of chicks moving). The occurrence of ‘Large scale movements’ during videos (> 50% of chicks moving between rather than within grid-squares) was a binary variable scored for the whole minute (yes/no). ‘Drinking’ was a count of chicks observed using nipple drinkers. As feed was scattered on the litter as well as available in hoppers, ‘Foraging’ was a count of chicks observed either with heads down/scratching or directly pecking at feeder pans. Numeric variables: Foraging (Pearson’s r = 0.32, t = 7.42, p < 0.0001), Drinking (r = 0.43, t = 10.64, p < 0.0001) and Total Chicks (r = 0.27, t = 6.10, p < 0.0001) were significantly correlated across and averaged for the Surrounding squares.

To ensure that comparable data was collected in all Flocks, video analyses were limited to 3 x 2m^2^ areas that contained the same arrangement of features without obstructions to visibility (by feeder/drinker lines or overhead fans, pillars etc). Visible floor space beyond these areas varied between Flocks due to the position of obstructions in different Houses and a slight tilt of the fish-eye lens that could alter how much floor space was captured as the extremes of the field of view. However, spearman rank correlations between spatial distribution/activity scored for all visible floor space in videos and the same variables scored for ‘surrounding’ squares suggest the 6m^2^ area observed was a representative sample of broader flock activity (distribution: Rho 0.51, p <0.0001; activity: Rho 0.80, p < 0.0001). Distribution and Activity were therefore classified for the two surrounding squares combined.

**Welfare and Productivity data**

To test whether early-life distress calling predicted immediate and future weight or mortality, farm productivity data were collated for the days following acoustic measurements (days 2-5 of placement) and day 32. Mortality (birds found dead, excluding culls) was collated from stockperson records. Average bird weight was provided by the producer, from a commercial algorithm using data collected continuously from two platform balances (Fancom Automatic Poulty Weighing System; Leuven, Belgium) in each house. Because data were collected on a commercial farm, ages for house thinning (25-30% flock slaughtered) and clearance varied slightly across flocks as required to optimise productivity (thinning 33-34 days; clearance 36-39 days). For consistency, end point data were gathered at 32 days to pre-date these events. Parameters collated at flock-level were therefore: mortality and average bird weight on days 2-5 post-placement, and % flock mortality and average bird weight on day 32 post-placement. To account for slight variation in numbers placed (25090-26510), mortality was expressed as a percentage of total placed, minus cumulative losses in the preceding days for day 2-5 data. Specific welfare markers that are routinely gathered at slaughter, such as pododermatitis and hock burn prevalence, accumulate with age thus could not be compared between flocks here due to variation in age at slaughter (36-39 days).

**Statistical methods**

The best automated proxy of manual distress call counts was assessed in three ways. First, the manual call count was skewed toward low values, so a Spearman’s correlation matrix of manual call count against all acoustic parameters was generated. Second, a random forest approach (R randomForest, Liaw and Wiener, 2002) was used for parameter selection. The model compared 2000 trees, each composed of subsets of 12 variables to reduce impacts of collinearity in parameter selection. Parameter ‘importance’ for this approach was measured as the difference in mean squared error with random permutation of each variable, normalized by the standard deviation in those differences. This approach is relatively robust to within-variable correlation and variation in scales of measurement across parameters (Nicodemus, 2011). Third, for the parameters with the strongest correlation, a linear mixed model (LME) was fitted for each with Flock as a random effect to account for repeated measurements, manual distress call count as the dependent variable and the interaction of day of placement (factor, 1-4) x the acoustic correlate as independent variables. A likelihood ratio test between models with and without the interaction was used to assess whether the slope of the correlation between this parameter and distress calling was age dependent. Thus, Spearman’s Rho, ranked Importance and age-independence were used to select the parameter with greatest predictive capacity (termed the ‘acoustic predictor’ for the remainder of the methods).

To determine whether changes in call rate reflected changes in flock-level behaviour, we then tested for relationships between the acoustic predictor and data from time-matched videos. Unsurprisingly, behaviour differed markedly on day 1 of placement. For example, while Total Chicks across the three squares was variable on all days (‘Total chicks’ coefficient of variation, c.v., day 1: 53%, day 2: 45%, day 3: 36%), chance positioning of a large cluster of birds in one square explained 66% and 57% of the top third of c.v. values on days 2 and 3 respectively, but only 21% of high c.v. values on day 1 where distribution was generally less even. As such, models were produced separately for day 1 (115 videos, 12 flocks) and days 2-3 (378 videos, 10 flocks). In both analyses, an LME fitted with Maximum Likelihood was used to control for repeated measurements per Flock as a random effect. Total chicks, Distribution, Foraging, Drinking and Activity for the Microphone square and Surrounding squares and the parameter Large-Scale Movements were independent variables in the models. In the day 1 model, hours from placement was also included as a covariate. Activity in surrounding squares had little variation on day 1 (no category 0s, 9 x 1s, 106 x 2s), which was captured by the Large-Scale Movement parameter (effectively Activity category 2 v Large-Scale movements) so was omitted from that model. The day 2-3 model included day of placement as a factor (2 or 3, categorical). Microphone and Surrounding square Total Chicks were retained in models to control for number of chicks near the microphone, but models were otherwise simplified by backwards stepwise regression, using Likelihood Ratio Tests (LRT) to compare consecutive models (threshold p < 0.05) until only significant variables and chick counts remained.

Finally, we tested whether the acoustic predictor, expressed as the median of the recordings collected per flock per day, was predictive of mortality and weight gain. On days 2-4, 144 recordings collected over 24 h were available but on day 1 placement time and hence hours of recording varied across flocks. As such, we calculated the median using 42 recordings collected over hours 1-8 of placement on day 1, which was the maximum duration available for the latest placed flock but censoring the first hour in case of ongoing stockperson disturbance associated with placement. In the first pair of models, the response variables were average bird weight and proportion flock mortality in the next day. As such, there were 4 flock-level mortality/weight measurements (day 2, 3, 4 and 5) per flock and LMEs were used to control for repeated measurements per flock, as above. The main effects and interaction of the acoustic predictor x Age were independent variables. Mortality was logged to improve model fit. In the second pair of models, to test whether early life distress calling predicted late life productivity and welfare, linear models were constructed with the response variable of either Mean Bird Weight or % Flock mortality at 32 days. In these models, there were 12 data points (one per Flock), and four independent variables: the average o the acoustic correlate per age 1-4 per flock. Models were simplified by stepwise backward regression until only significant variables remained.

Sound was off during video data extraction, and acoustic, video and welfare data were compiled separately so that researchers would remain blind to outcomes during manual data extraction.

SUPPLEMENTARY FIGURES AND TABLES


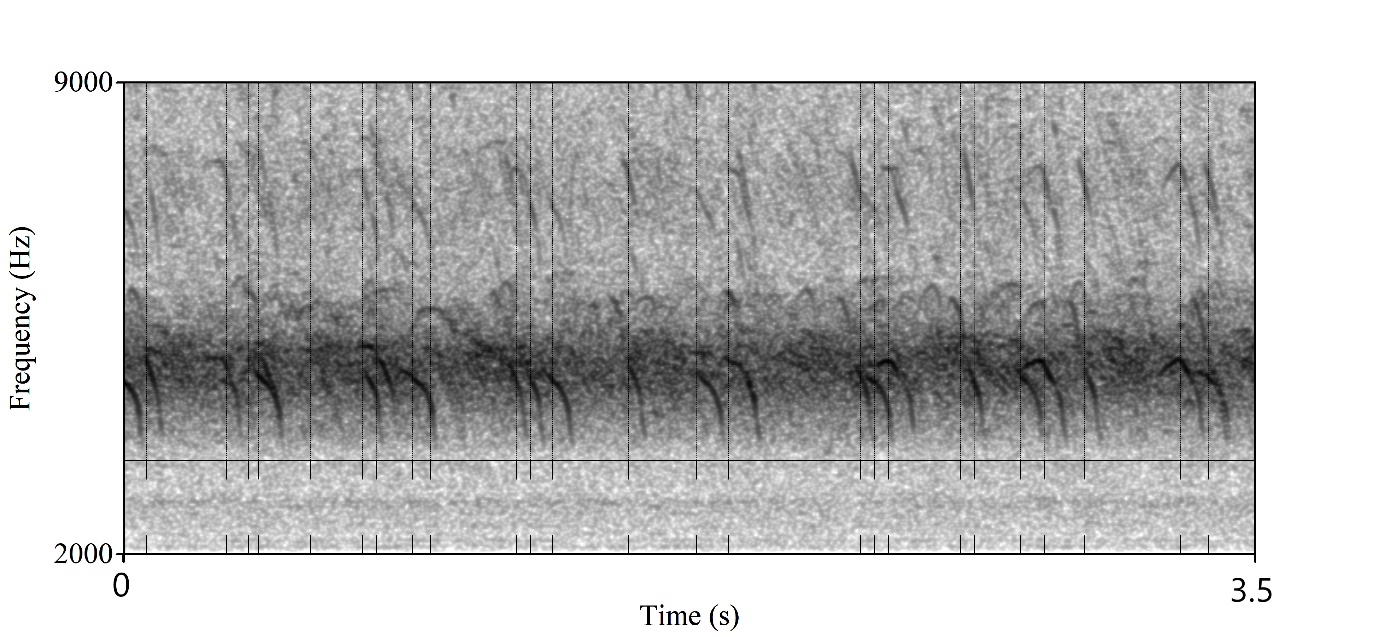


**Figure S1 3.5s spectrogram with 0.03s window-length and 70dB dynamic range (Praat, Boersma, 2001), to illustrate manual count of 25 visible distress calls indicated with lines.**


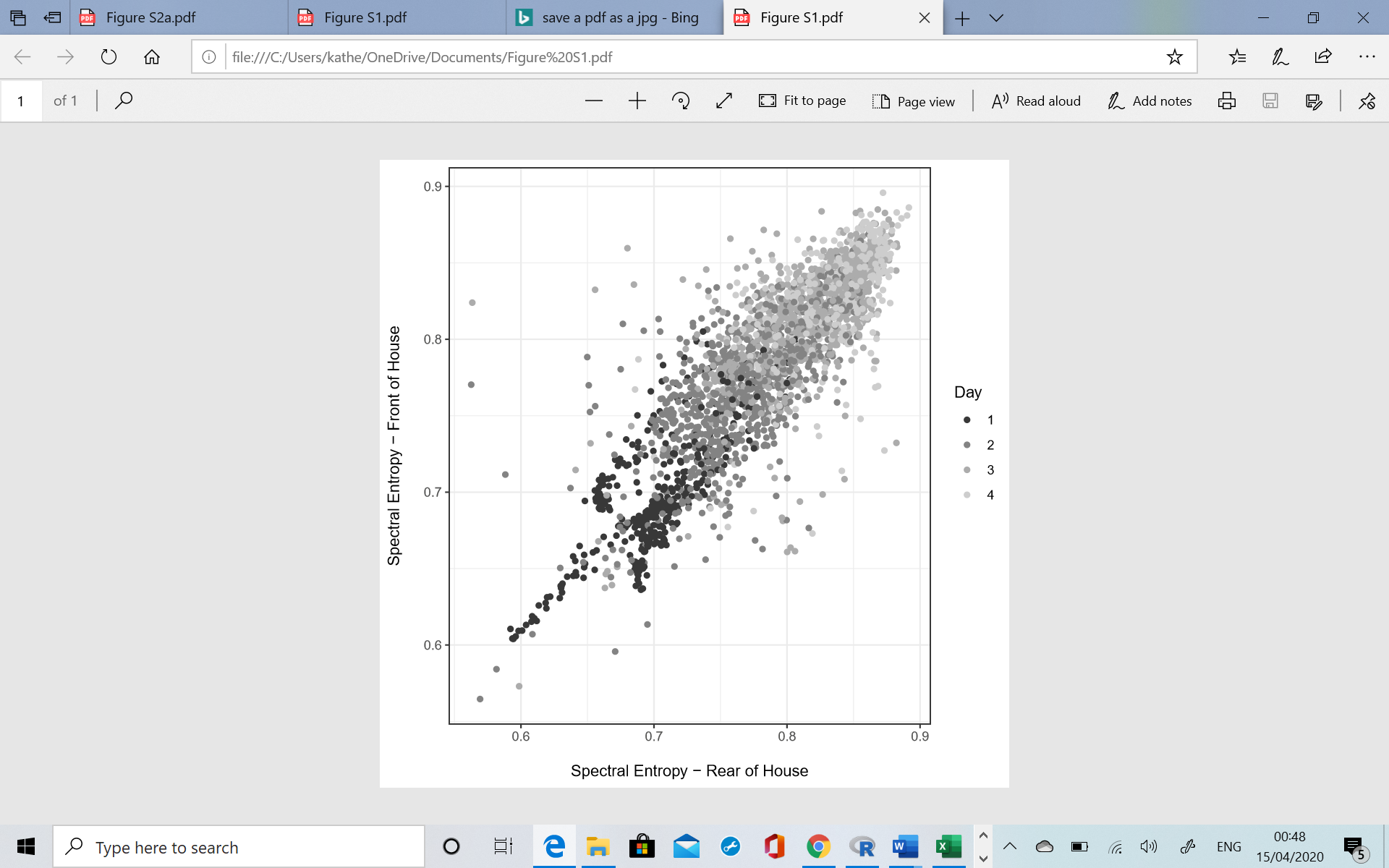


**Figure S2 High-pass filtered Spectral Entropy in time-matched 1-minute recordings from the front versus rear of house on day 1-4s of placement. N = 2584, 6 Flocks.**

a) b)


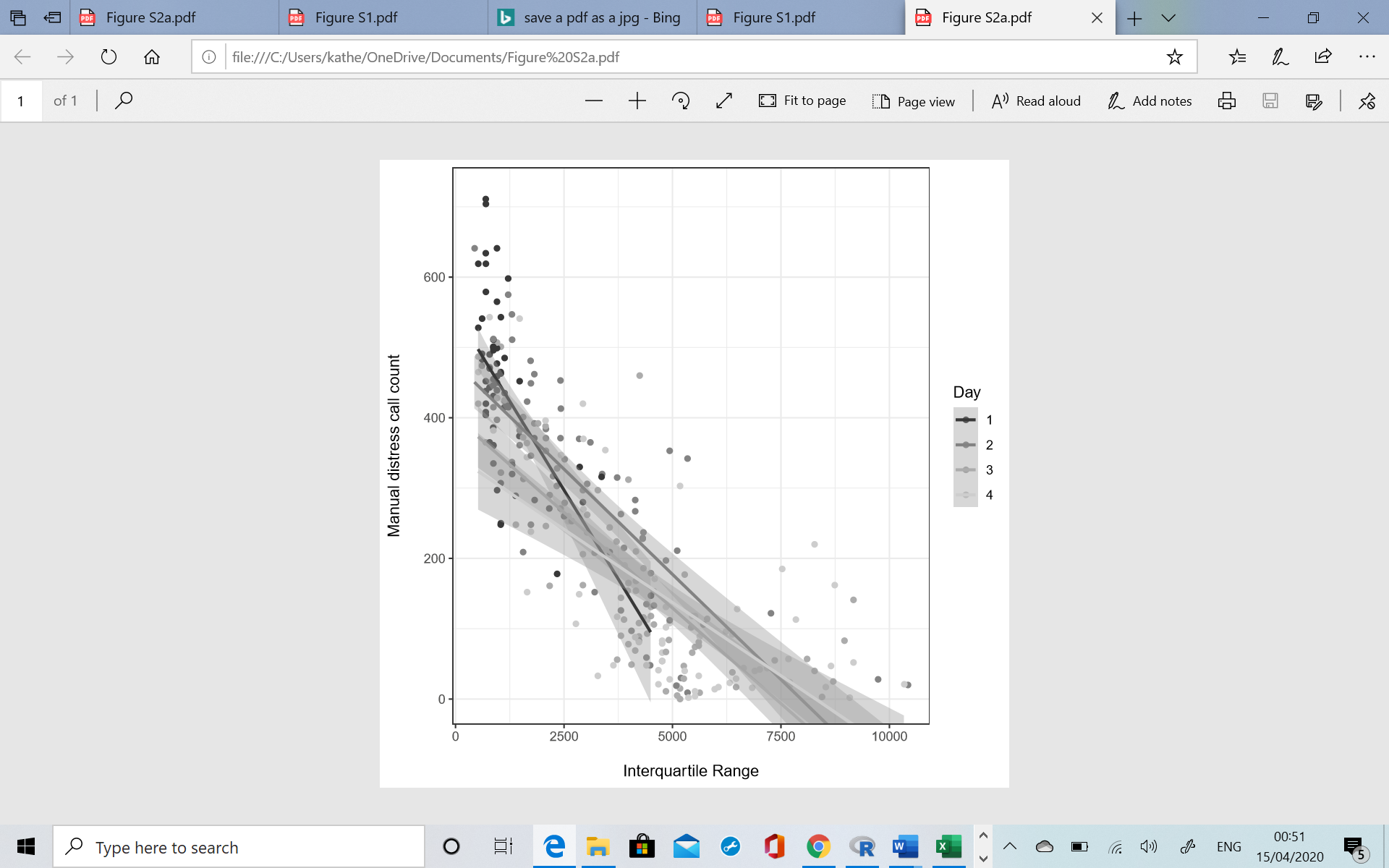

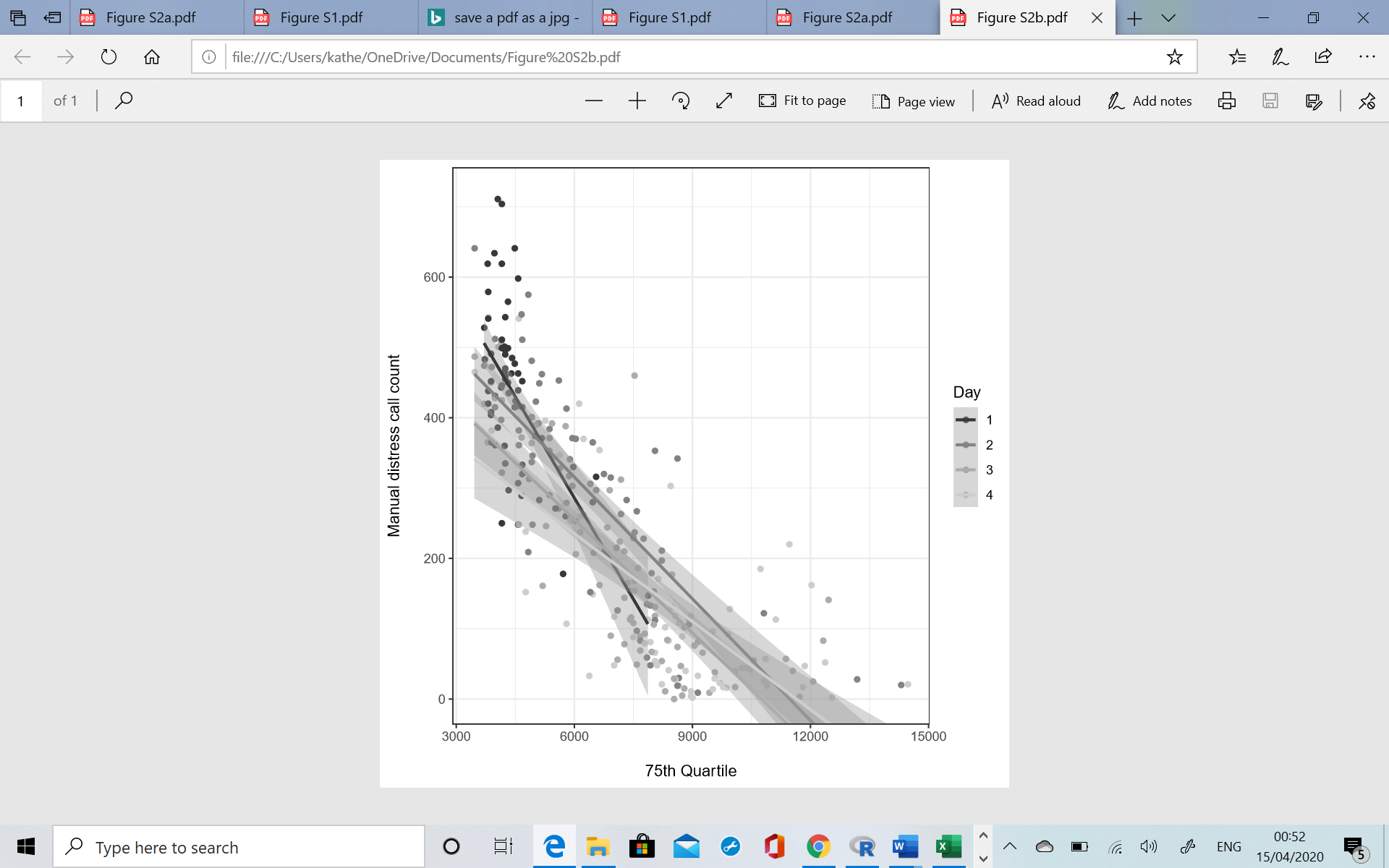


**Figure S3 Relationship between manual distress call count and a) interquartile range and b) 75^th^ Quartile of high-pass filtered frequency distributions. Point/line shade indicates day of placement, shaded area indicates confidence interval.** **N = 283 datapoints from 12 Flocks**.

**Table S1 Linear mixed models of manual distress call count with the interaction of Day x a) the Interquartile range and b) 75^th^ Quartile of the frequency distribution. N = 283/12 Flocks.**

| **Parameter** | **Coefficient** | **Std. Error** | **DF** | **t-value** | **p-value** |
| --- | --- | --- | --- | --- | --- |
| *a) Interquartile range* |  |  |  |  |  |
| Intercept | 547.18 | 22.23 | 264 | 24.61 | <0.0001 |
| IQR (Hz) | -0.10 | 0.01 | 264 | -6.78 | <0.0001 |
| Day of Placement – 2 | -64.54 | 29.11 | 264 | -2.22 | 0.028 |
| Day of Placement – 3 | -141.24 | 31.85 | 264 | -4.43 | <0.0001 |
| Day of Placement – 4 | -184.03 | 34.46 | 264 | -5.34 | <0.0001 |
| IQR x Day of Placement – 2 | 0.04 | 0.02 | 264 | 2.37 | 0.019 |
| IQR x Day of Placement – 3 | 0.04 | 0.02 | 264 | 2.79 | 0.0057 |
| IQR x Day of Placement – 4 | 0.05 | 0.02 | 264 | 3.46 | 0.0006 |
| *b) 75th Quartile* |  |  |  |  |  |
| Intercept | 854.65 | 65.72 | 264 | 13.00 | <0.0001 |
| 75th Quartile (Hz) | -0.09 | 0.01 | 264 | -6.61 | <0.0001 |
| Day of Placement - 2 | -182.37 | 74.65 | 264 | -2.44 | 0.015 |
| Day of Placement - 3 | -272.28 | 76.35 | 264 | -3.57 | 0.0004 |
| Day of Placement - 4 | -350.99 | 78.31 | 264 | -4.48 | <0.0001 |
| IQR x Day of Placement - 2 | 0.04 | 0.02 | 264 | 2.30 | 0.022 |
| IQR x Day of Placement - 3 | 0.04 | 0.02 | 264 | 2.64 | 0.0088 |
| IQR x Day of Placement - 4 | 0.05 | 0.02 | 264 | 3.33 | 0.001 |

REFERENCES

Aide, T.M., Corrada-Bravo, C., Campos-Cerqueira, M., Milan, C., Vega, G., Alvarez, R. 2013. Real-time bioacoustics monitoring and automated species identification. PeerJ 1: e103

Boersma, P. 2001. Praat, a system for doing phonetics by computer. Glot International 5: 341-345.

Fontana, I., Tullo, E., Scrase, A., Butterworth, A. 2016. Vocalisation sound pattern identification in young broiler chickens. Animal 10: 1567-1574

Liaw, A., Wiener, M. 2002. Classification and Regression by randomForest. R News 2: 18-22

Ligges, U., Krey, S., Mersmann, O., Schnackenberg, S. 2018. tuneR: Analysis of Music and Speech. URL: https://CRAN.R-project.org/package=tuneR

Marx, G., Leppelt, J., Ellendorff, F. 2001. Vocalisation in chicks (Gallus gallus dom.) during stepwise social isolation. Applied Animal Behaviour Science 75: 61-74

Nicodemus, K.K. 2011. Letter to the Editor: On the stability and ranking of predictors from random forest variable importance measures. Briefings in Bioinformatics 12: 369–373

ROSS Broiler Management Handbook. 2018. Huntsville: Aviagen

Sueur, J., Aubin, T., Simonis, C. 2008. Seewave: a free modular tool for sound analysis and synthesis. Bioacoustics 18: 213-226.
